# Neutrophil extracellular traps in gout: from immune defense to pathological dynamic equilibrium

**DOI:** 10.3389/fimmu.2026.1826191

**Published:** 2026-05-13

**Authors:** Fangfang Wang, Ning Tie, Yong Jin, Hongbin Li

**Affiliations:** 1 Department of Rheumatology and Immunology, Inner Mongolia Medical University, Hohhot, China; 2 Inner Mongolia Key Laboratory for Pathogenesis and Diagnosis of Rheumatic and Autoimmune Diseases, Department of Rheumatology and Immunology, Affiliated Hospital of Inner Mongolia Medical University, Hohhot, China

**Keywords:** gout, inflammatory response, monosodium urate crystals, neutrophil extracellular traps, therapeutic targets

## Abstract

Gout is an aseptic inflammatory disease caused by monosodium urate (MSU) crystal deposition. Its clinical signs go beyond the intense pain of acute arthritis to include structural damage such as chronic synovitis, bone erosion, and tophus formation. The long-term progression and recurring episodes of inflammation pose significant challenges in managing the disease. While hyperuricemia creates the metabolic basis, only a small percentage of patients develop gout, indicating that activation of the innate immune system is essential for its development. Recently, neutrophil extracellular traps (NETs) have become recognized as key mediators connecting metabolic issues to inflammatory responses, representing an important breakthrough in understanding gout pathogenesis. Initially, NETs are considered a host defense mechanism that protects against microbial invasion, where neutrophils release a web-like structure made of chromatin fibers and granular proteins to trap and eliminate microbes. However, in gout, a non-infectious disease, NETs have roles that extend beyond their traditional function, acting as a “double-edged sword”: they help limit acute inflammation but can also contribute to tissue damage and disease progression during the chronic phase, with their function changing according to the microenvironment. This review systematically discusses the mechanisms of NETs in gout development and examines their potential in diagnosing and treating the disease.

## 1 Introduction

Gout is a common metabolic disorder. Its core pathogenesis involves the deposition of monosodium urate (MSU) crystals in joints and surrounding tissues due to hyperuricemia, thereby triggering acute inflammatory responses (1). The pathogenesis of hyperuricemia is complex and multifaceted, involving increased uric acid production, impaired renal and intestinal excretion, and genetic polymorphisms in uric acid transporters (1). Notably, although most hyperuricemic individuals do not develop gout, their persistent low-grade inflammatory state significantly elevates the risk of cardiovascular and metabolic diseases (2, 3). Acute gout attacks feature aberrant activation of the innate immune system, particularly the NOD-like receptor family pyrin domain-containing 3 (NLRP3) inflammasome. This activation facilitates the conversion of precursor interleukin-1β (pro-IL-1β) into bioactive interleukin-1β (IL-1β), which in turn drives the recruitment and activation of inflammatory cells and elicits severe local inflammation (1, 4). Uncontrolled hyperuricemia leads to recurrent MSU crystal deposition, which drives disease chronicity and results in damaged joint structure and tophus formation (5).

Neutrophil extracellular traps (NETs) are reticular structures composed of disaggregated chromatin and granular proteins. Neutrophils release these structures in response to pathogen- or inflammation-associated signals, which trap foreign substances such as bacteria, fungi, and crystals to limit their spread. Functional proteins attached to DNA, including histones, myeloperoxidase (MPO), and neutrophil elastase (NE), eliminate pathogens through direct killing or enzymatic degradation, forming a crucial defense in the innate immune system (6–8). Recent research has revealed that NETs extend beyond anti-infectious functions and contribute to the pathogenesis of diverse non-infectious inflammatory disorders, including autoimmune diseases, cardiovascular diseases, cancer, and metabolic diseases (9–14). Researchers have not yet fully elucidated their direct role in gout-related bone erosion and chronic synovitis. Could NETs drive an “autoimmune-like” response? It has been demonstrated that MSU crystals are a key stimulus for neutrophil NET release (15), and clinicians detect these crystals in the serum and synovial fluid of patients with gout (16). Notably, NETs display a distinct “double-edged sword” role: on one hand, NETs facilitate the spontaneous resolution of acute inflammation by sequestering MSU crystals, degrading inflammatory mediators, and restricting excessive neutrophil infiltration (15, 17); on the other hand, NET components such as histones and NE exacerbate joint inflammatory damage and promote tophus formation in the chronic phase, thereby driving disease chronicity and structural destruction (18). In this review, we systematically elucidate the dual roles and underlying molecular mechanisms of NETs in gout pathogenesis by characterizing their formation and functional properties, summarize their potential clinical utility for gout diagnosis and treatment, and propose the precise regulation of NET homeostasis as a novel therapeutic strategy for gout.

## 2 Formation and clearance mechanisms of NETs

### 2.1 Formation of NETs

Neutrophils release NETs—reticular structures formed by the interweaving of DNA fibers—when activated, and researchers refer to the active release process as NETosis. As a unique cellular response specific to neutrophils, NETosis differs significantly from necrosis and apoptosis. Activated neutrophils form reticular structures composed of nuclear components and granular substances, which ultimately aggregate into linear structures with a diameter of 50 nm (7, 19). Takei et al. first reported a novel suicide-like death mode of neutrophils in 1996 (20); this process takes several hours and features a series of progressive changes, including gradual chromatin decondensation, nuclear swelling, leakage of nucleoplasm into the cytoplasm, and cell membrane perforation. Brinkmann et al. first discovered and characterized NETs in 2004 via immunofluorescence and DNA staining techniques (7), thereby establishing a foundational framework for subsequent investigations.

The core components of NETs are histones and highly decondensed chromatin fibers, with a diameter of 15~17 nm (7, 21). Core histones include four subtypes: H2A, H2B, H3, and H4. Granular components account for 70% of NET-related proteins, form spherical structures with a diameter of 25 nm, and are stored primarily in neutrophil-specific granules attached to the NET DNA backbone. Various functional enzymes—including NE, MPO, cathepsin G, leukocyte protease 3, lactoferrin, gelatinase, lysozyme C, and calprotectin—mediate the efficient antibacterial effects of NETs (7, 22). Under physiological conditions, neutrophils mainly produce NETs to respond to various microbial infections, including bacteria (23), fungi (22), and viruses (24). At the site of inflammation, NETs capture and trap pathogens through physical effects, while promoting the synthesis of antibacterial proteins and the recruitment of phagocytes to enable effective pathogen clearance (25). In addition, a variety of inflammatory signals can induce NET formation during the pathological process of non-infectious diseases. At present, the scientific community has not yet fully elucidated the specific molecular mechanisms and signaling pathways by which NETs exert their roles in various diseases. However, their unique structural and functional characteristics have become a major focus of research. An in-depth investigation into the mechanisms governing NET formation and their key regulatory factors is poised to identify novel therapeutic targets and research avenues for the clinical management of infectious and non-infectious inflammatory disorders.

**Figure 1:**
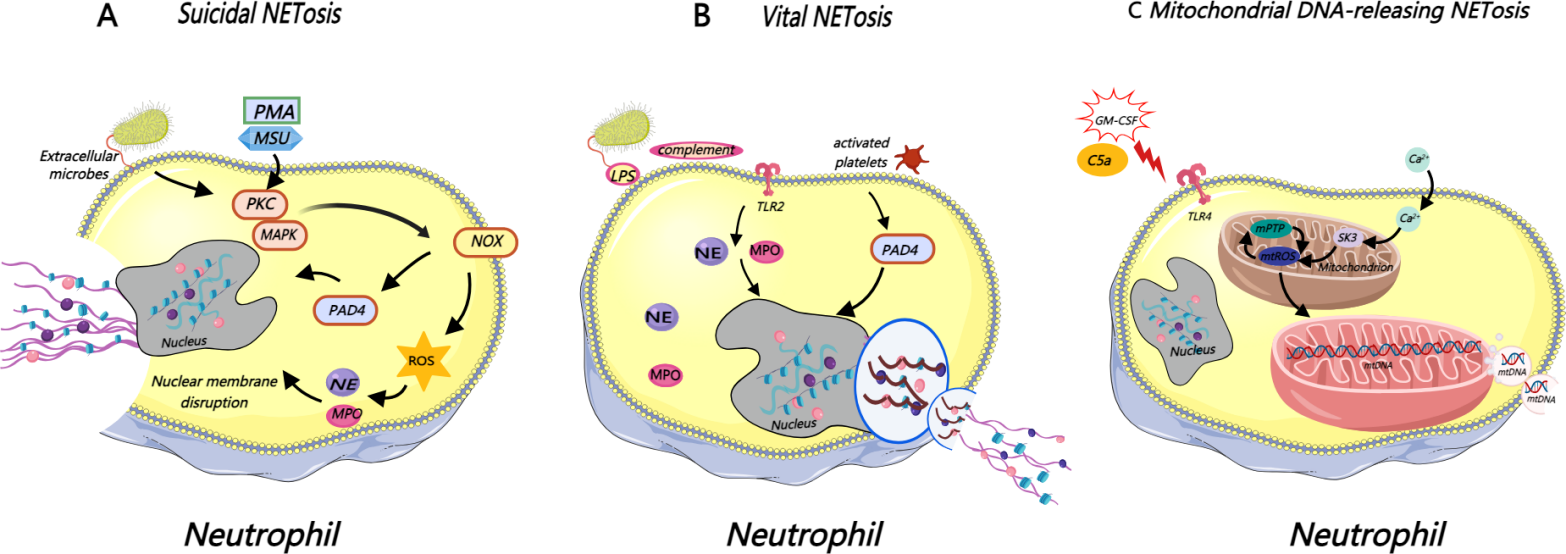
Classification of NETosis into suicidal, vital, and mitochondrial DNA‑releasing modes.

It has been demonstrated that NETosis can be classified into three main modes: suicidal NETosis, vital NETosis (7), and mitochondrial DNA-releasing NETosis (26) (**Figure 1**). Classical suicidal NETosis centers on a NADPH oxidase (NOX)-mediated reactive oxygen species (ROS) burst as the core upstream event. This regulatory pathway proceeds as follows: ROS activate key signaling pathways, including protein kinase C (PKC) and mitogen-activated protein kinase (MAPK), which in turn activate peptidylarginine deiminase 4 (PAD4). PAD4 disrupts chromatin structure stability through the catalysis of histone citrullination, leading to chromatin decondensation. Ultimately, the nuclear and cytoplasmic membranes rupture sequentially, and neutrophils release a complex mixture of chromatin and cytoplasmic granular proteins, which assemble to generate NETs (27). Vital NETosis represents a non-death NET release mechanism of neutrophils. Factors such as the bacterial product LPS, activated platelets, and complement activation can induce this process, which is independent of the ROS pathway. Neutrophils retain some physiological functions, such as chemotaxis and phagocytosis, after rapidly releasing NETs (27). Pilsczek et al. first observed this mode in 2010 under stimulation with *Staphylococcus aureus* (23): neutrophils undergo nuclear swelling and form vesicles within 5~60 minutes, which then fuse with the cell membrane to release NETs after carrying DNA. This process does not require cell lysis, is independent of ROS, and depends on TLR2 and complement component C3; the released NETs mainly consist of nuclear DNA. This “rapid release” mechanism—vital NETosis—results in a much faster NET formation rate than the previously recognized “chronic process”. Neutrophils that preserve their nuclear structure following NET release retain both biological activity and migratory capacity within tissues. Vital NETosis has broken the traditional cognition that “NETosis necessarily accompanies cell lysis” (28). In gout-related studies, researchers have found scarce relevant evidence on the induction of non-death NET release in neutrophils.

Mitochondrial DNA-releasing NETosis also represents a non-death NETosis. Yousefi et al. demonstrated in a 2009 study that viable neutrophils generate mtDNA-based NETs (instead of nuclear DNA-based NETs) following pretreatment with granulocyte-macrophage colony-stimulating factor (GM-CSF) and subsequent stimulation via Toll-like receptor 4 (TLR4) or complement factor 5a (C5a) receptors (29). Furthermore, this process does not require neutrophil death, does not shorten cell lifespan, and neutrophils producing such NETs even exhibit a higher survival rate than resting neutrophils that do not produce NETs (29). Subsequent studies have further confirmed that mitochondrial NET formation also depends on ROS, which the mitochondrial pathway mainly produces (30). David et al. clarified the key regulatory mechanism of this mode in 2015 (31): calcium-induced SK3 channel activation and mitochondrial ROS (mitoROS) production jointly trigger the opening of the mitochondrial permeability transition pore (mPTP), which in turn leads to the loss of mitochondrial membrane potential and mitochondrial swelling, ultimately promoting the release of mitochondrial DNA (mtDNA) to form NETs.

### 2.2 Formation and regulation of NETs induced by MSU crystals

As the core pathogenic factor in the progression of gout, MSU crystals potently induce ROS-dependent NETosis (32). Evidence suggested that MSU crystals not only induce NET formation in neutrophils (33, 34) but also serve as an initiating signal that remodels their functional phenotype. The molecular mechanism by which MSU crystals regulate neutrophil activation and NET formation features a multi-node, network-based core. The interaction between the crystals and the neutrophil surface initiates MSU crystal-mediated neutrophil activation (35), a process that relies on a “multi-node cross-activation” molecular network. This network centers on pattern recognition receptors (PRRs) and cooperates with multiple downstream pathways—including autophagy, inflammasome activation, and kinase signal transduction—to form a regulatory network. The PRRs driving the initiation of this signaling network primarily include Toll-like receptors 2 and 4 (TLR2/4), scavenger receptor A (SRA), and the purinergic receptor P2Y6. Various receptors work synergistically to trigger the initial activation of neutrophils (36–39).

First, MSU crystals specifically bind to TLR-2/4 on the neutrophil surface, recruit and activate myeloid differentiation factor 88 (MyD88), and then activate the nuclear factor-κB (NF-κB) pathway through MyD88-dependent signal transduction. NF-κB pathway activation significantly drives the transcription and expression of early inflammatory cytokines such as tumor necrosis factor-α (TNF-α), further establishing and maintaining an inflammatory microenvironment that lays the foundation for subsequent NET formation (37). Meanwhile, activated neutrophils upregulate PAD4 expression via the ATG7-p53 signaling pathway. This process further potentiates the histone citrullination process described in Section 2.1 and accelerates subsequent NET formation (40). Second, SRA-mediated endocytosis of MSU crystals can disrupt lysosomal membrane stability. The release of its contents not only exacerbates inflammation but also selectively activates the ERK signaling pathway and significantly increases intracellular ROS levels (38), driving classical ROS-dependent NETosis (41, 42). Upon contact with or phagocytosis of MSU crystals, neutrophils release extracellular uridine diphosphate (UDP), which acts as a high-affinity ligand for P2Y6 receptors. UDP then binds to surface P2Y6 receptors, resulting in calcium influx and further triggering the classical ROS pathway (39). As a classic “danger signal”, MSU crystals effectively activate the NLRP3 inflammasome, resulting in the maturation and secretion of IL-1β. IL-1β binds to IL-1R on the neutrophil surface in an autocrine/paracrine manner, further amplifying signals such as MAPK and forming a self-reinforcing inflammatory loop that continuously promotes NET formation (15, 43).

**Figure 2:**
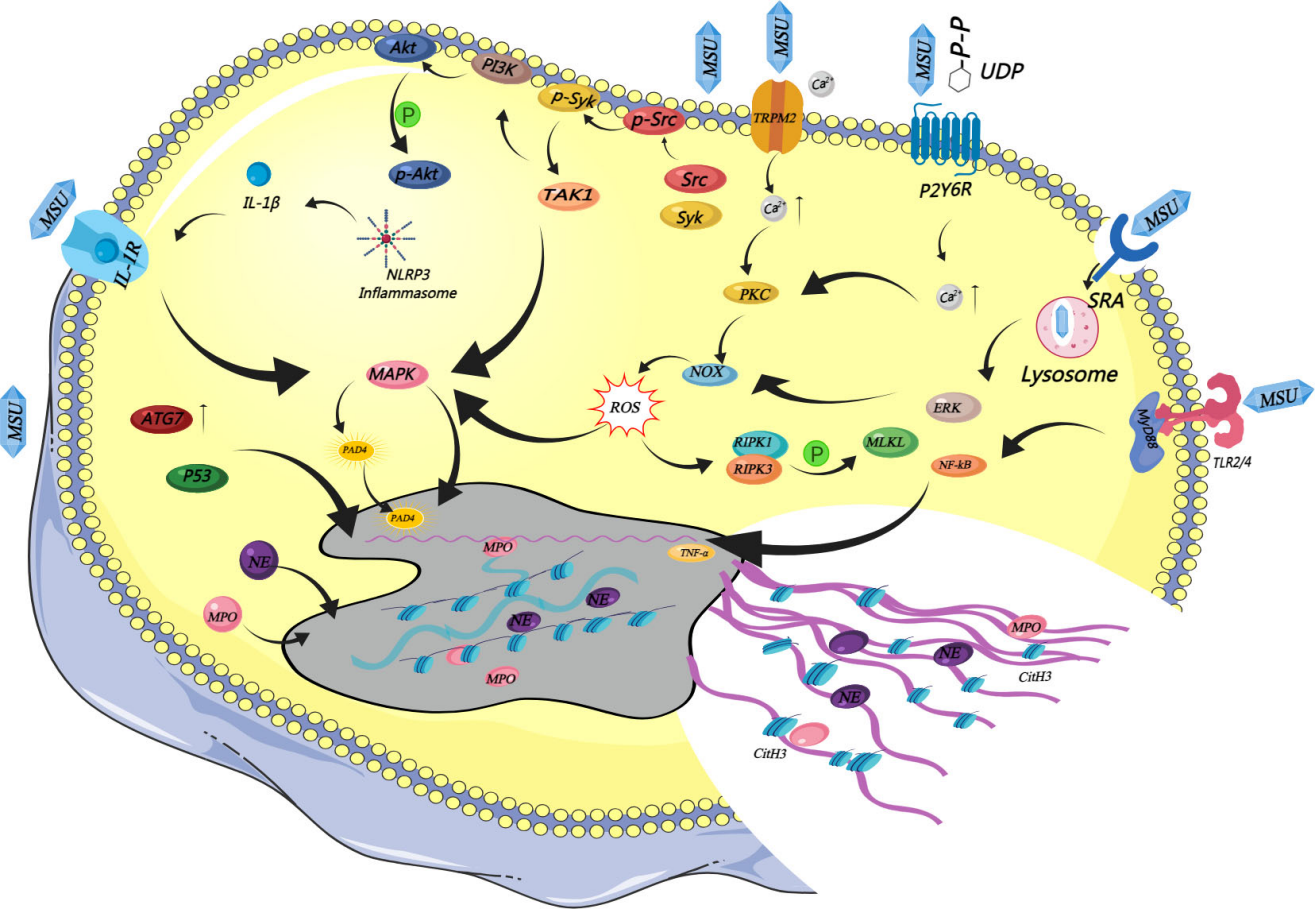
Alternative and complementary signaling pathways regulating MSU-induced NETosis.

However, whether MSU crystal-induced NETosis is strictly dependent on ROS remains a key controversy in this field, and current research findings exhibit considerable divergence. Numerous *in vitro* studies using isolated neutrophils support that NETosis is highly dependent on ROS production, which is generated by either the NOX or mitochondrial pathway, leading to NOX-dependent or NOX-independent NETosis, respectively (44). Wu et al. found that NOX inhibitors can significantly inhibit MSU crystal-induced NET formation (41). Cao et al. demonstrated that transient receptor potential melastatin 2 (TRPM2), a calcium-permeable cation channel, first mediates intracellular calcium influx to activate the PKC-NADPH oxidase pathway upon MSU crystal stimulation of neutrophils, thereby promoting ROS production (45). Activation of this pathway constitutes a necessary condition for MSU-dependent NOX-induced NETosis. These findings collectively verify the classical “MSU crystal-ROS-NETs” signaling axis. Davidsson et al. found that neutrophils derived from the skin of patients with gout exhibit unique NET-producing characteristics. These cells can still efficiently generate NETs even when researchers inhibit NADPH oxidase (46, 47). This phenomenon indicates that the *in vivo* pre-sensitized state of neutrophils modulates their NETosis signaling pathways, allowing these cells to bypass the canonical ROS pathway and instead generate and release NETs via alternative NOX-independent signaling pathways. Dhia et al. found that ultraviolet irradiation of mitochondria can induce NOX-independent NETosis (48). Current studies have also demonstrated that MSU-induced NETosis is ROS-independent, and pharmacological inhibition of PAD4 potently abrogates this process (49). After the interaction between MSU and neutrophils, Src and Syk kinases rapidly activate. Upstream TAK1 and Syk serve as core regulatory molecules, further mediating the phosphorylation and activation of PI3K-Akt and MAPKs. This signaling axis not only regulates neutrophil chemokine production but also acts as a key alternative pathway mediating MSU-induced NETosis (49). In the case of NADPH oxidase inhibition, this pathway can complement the classical ROS pathway to support the efficient production of NETs in neutrophils of gout patients. In addition to the above alternative pathways, the formation mechanism of NETs also involves a broader regulatory network. Evidence suggests that the classical NETosis pathway mainly relies on key molecules such as ROS, PAD4, PKC, and MAPK. In recent years, studies have found that MSU crystal-induced neutrophil death and NET formation may also involve the necroptosis signaling pathway downstream of ROS production. ROS first mediates this pathway to activate receptor-interacting protein kinase 1 (RIPK1), which then recruits and binds RIPK3 to form a complex. Subsequently, mixed lineage kinase domain-like protein (MLKL) undergoes phosphorylation, which then translocates to the nuclear membrane and plasma membrane, ultimately triggering the rupture of these membranes and mediating NET formation. The regulatory mechanism centered on RIPK1, RIPK3, and MLKL provides a new potential direction for the molecular targeted therapy of crystal-related diseases such as gout (50) (**Figure 2**).

**Figure 3:**
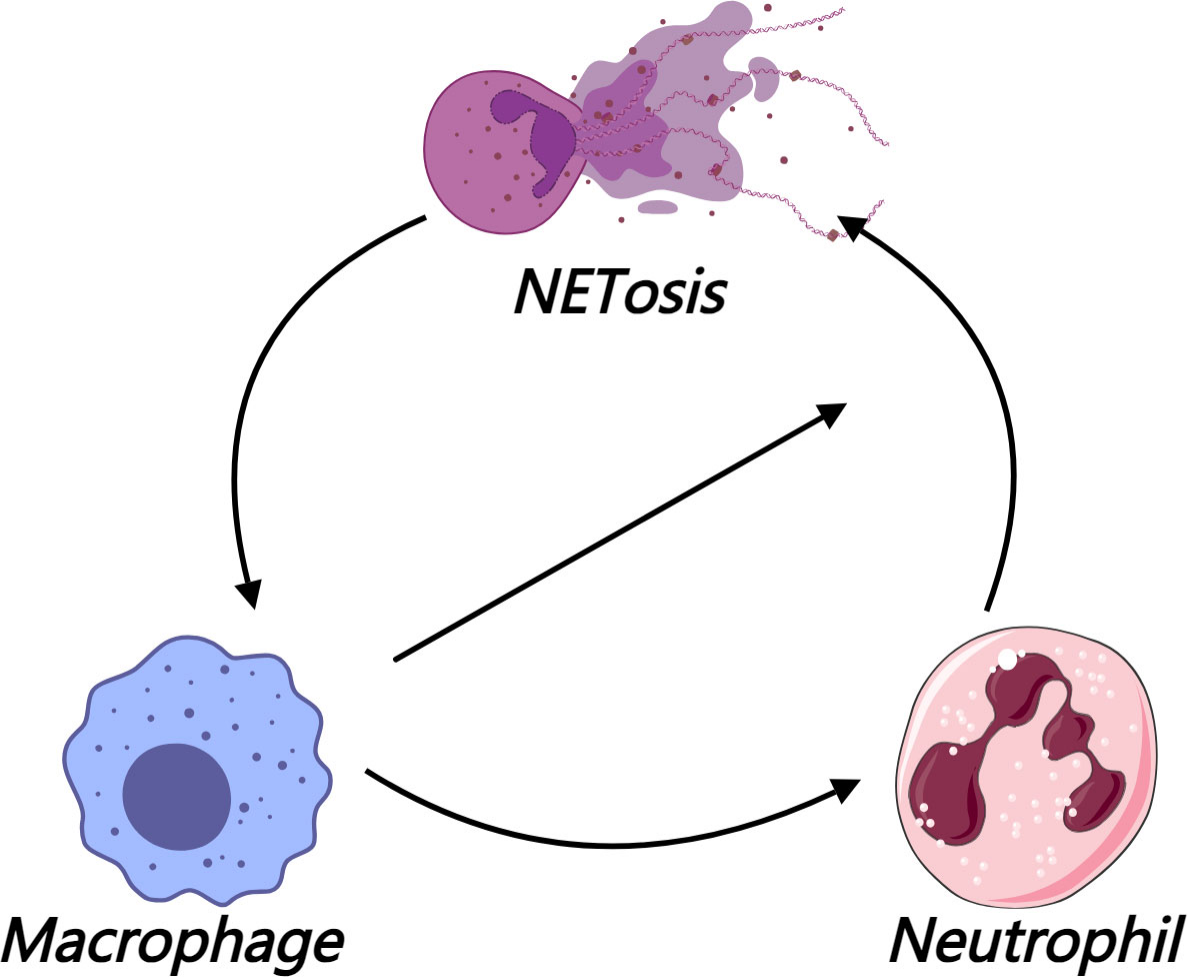
The "macrophage-neutrophil-NETs" feedback loop in gouty inflammation.

More importantly, NETs can reshape the inflammatory microenvironment through a complex immune interaction network, particularly through the bidirectional regulation between NETs and macrophages. On the one hand, NETs drive the polarization of macrophages toward the inflammatory M1 phenotype via a hexokinase-2 (HK2)-dependent molecular mechanism. M1 macrophages highly express inflammatory factors such as IL-1β and TNF-α, further recruiting neutrophils (43). On the other hand, M1-type macrophages, in turn, upregulate caspase-11 expression through IL-1R signaling, which mediates cytokine storms and enhances the sensitivity of neutrophils to chemokines (51), further promoting NET release and forming a “macrophage-neutrophil-NETs” cycle (**Figure 3**).

### 2.3 Clearance mechanisms of NETs

Under physiological conditions, the body maintains NET homeostasis through precise clearance mechanisms. However, excessive NET formation, impaired degradation, or dysregulated NET turnover leads to aberrant local NET accumulation, which impairs host immune defense, amplifies inflammatory responses, triggers cytokine storms, and induces widespread tissue and organ damage. Ultimately, these events drive the development and progression of pathological inflammation and autoimmune disorders. The progression of numerous diseases—including systemic lupus erythematosus (52), rheumatoid arthritis (53), gout (43), psoriasis (54), and cancer (55)—is closely associated with this process. Notably, impaired NET clearance represents a core pathological mechanism driving structural joint damage in gout.

The body primarily relies on two pathways for the physiological clearance of NETs to maintain homeostatic balance: the first is enzymatic degradation, where serum deoxyribonuclease I (DNase I) directly hydrolyzes the DNA backbone of NETs to disrupt these extracellular traps structurally (12); the second is phagocytic clearance, where macrophages recognize and engulf NET debris via scavenger receptors and mediate their intracellular degradation (56). Nevertheless, the synovial microenvironment of gouty joints significantly suppresses both clearance pathways. MSU crystals directly impair lysosomal function in macrophages, thereby markedly reducing their capacity for NET phagocytosis and clearance (34, 56). Moreover, persistent chronic inflammation in the joint leads to decreased DNase I activity or the production of endogenous inhibitory factors, blocking the enzymatic degradation of NETs (12). Inhibition of these two clearance mechanisms ultimately leads to the sustained accumulation of NETs and their toxic components within the joint microenvironment, which creates a persistent inflammatory stimulus that drives chronic synovial inflammation. Furthermore, the continuous accumulation of NETs promotes tophus formation via a “structural scaffold” mechanism (detailed in Section 3.2.1), further exacerbating structural joint damage (18).

## 3 Role of NETs in gout pathogenesis

### 3.1 Role of NETs during acute gout attacks

During the initial stage of acute gout flare, NET formation reflects an immunological balance, with its role dynamically shifting between “inflammation restriction” and “inflammation amplification”; the outcome of this balance determines disease progression. In the early phase of acute inflammation, on one hand, the explosive formation of NETs may transiently overwhelm clearance mechanisms and establish a positive feedback loop with the complement system: NET-derived DNA activates complement to generate C5a, which in turn chemoattracts and activates additional neutrophils to release NETs, thereby rapidly amplifying inflammation (57). Free DNA within NETs also acts as a damage-associated molecular pattern (DAMP) to activate the absent in melanoma 2 (AIM2) inflammasome in synovial fibroblasts, inducing pyroptosis and the robust release of IL-1β and IL-18, forming a positive feedback loop of “NETs–AIM2–pyroptosis” that continuously amplifies local inflammation (58). On the other hand, neutrophils recruit into the joint cavity and undergo NETosis upon encountering MSU crystals at low cell density. NETs released at this stage, along with their associated proteases, histones, and other bioactive components, not only exacerbate tissue damage but also trigger the release of chemokines and inflammatory mediators that further propagate inflammatory cascades, thereby acting as a key inflammatory mediator.

**Figure 4:**
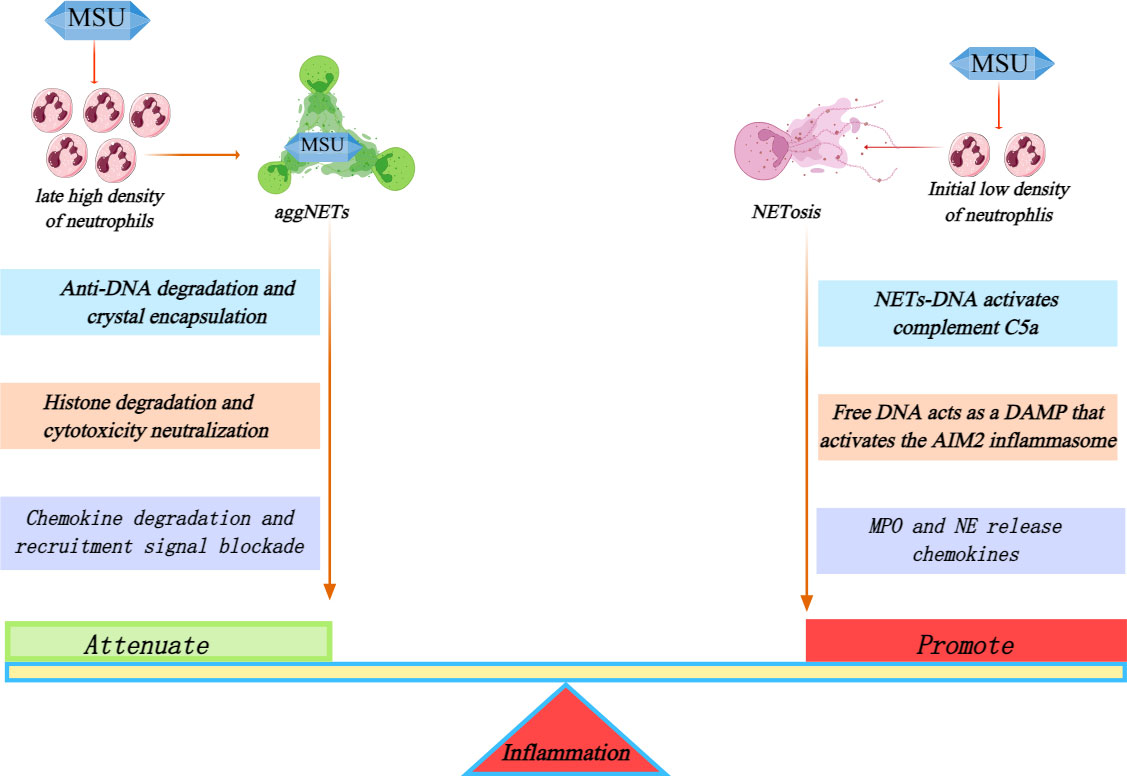
Opposing functions of NETs during acute gout attacks: inflammation amplification at low neutrophil density and inflammation resolution via aggNETs at high neutrophil density.

However, as inflammation advances and massive neutrophil infiltration leads to high cell density, the situation undergoes a fundamental shift. MSU crystal stimulation can induce the formation of aggregated NETs (aggNETs) (59). These aggNETs are rich in actin scaffolds, exhibit strong resistance to DNase-mediated degradation, and can entrap MSU crystals to prevent their dissemination (60). This process establishes an “immunologically silent” state, blocking persistent contact between crystals and immune cells and restricting inflammatory spread (61). Furthermore, studies have demonstrated that aggNETs degrade extracellular histones via their intrinsic MPO, and this neutralizes the cytotoxicity of histones to prevent excessive inflammatory propagation (59). Additionally, NE within aggNETs selectively degrades the chemokines IL-8 and CXCL5, terminating sustained recruitment signals and thereby exerting negative feedback regulation in the late phase of acute attacks (62) (**Figure 4**).

Regarding NET formation during this stage, colchicine—a widely used clinical agent—directly blocks NET release by interfering with F-actin polymerization (63), a key mechanism underlying its anti-inflammatory effects in acute gout flares. Clinical observations have shown that the proportion of aggNETs in synovial fluid positively correlates with the rate of spontaneous inflammatory resolution, supporting aggNETs as a critical molecular basis for the self-limited nature of gout (64). Current studies have clearly delineated the dual roles of NETs, yet researchers poorly understand the core regulatory nodes governing their functional transition. A dynamic threshold—determined by the collective effects of local MSU crystal concentration, NET formation rate, and host clearance efficiency—likely governs this phenotypic switch. When the rate of NET production exceeds the host’s degradative capacity, NET function shifts from controlled host defense to uncontrolled tissue damage.

### 3.2 Role of NETs in the chronic phase of gout

During the chronic phase of gout, the persistent accumulation and impaired clearance of NETs transform them from “dynamic regulators of homeostasis” in the acute stage into central drivers of structural joint damage and tophus formation.

#### 3.2.1 Mechanisms of NETs in tophus formation

During acute gouty inflammation, when the local neutrophil density within the joint reaches a critical threshold, MSU crystals trigger extensive neutrophil death and subsequent NET release. The released NETs can further aggregate to form three-dimensional mesh-like aggNETs (64). On one hand, aggNETs directly capture MSU crystals via physical sequestration. At high concentrations, aggNETs also overcome the host’s endogenous protease inhibitory system via neutrophil serine proteases (NSPs), including NE and MPO, thereby degrading inflammatory cytokines (e.g., IL-1β and TNF-α) and chemokines, which promotes the resolution of acute inflammation (64). On the other hand, as described earlier, aggNETs encase MSU crystals via the “immunological silencing” mechanism, forming the structural foundation for tophus deposition (61). In this state, MSU crystals cannot trigger new inflammatory responses, and the aggregates formed by aggNET-mediated entrapment and embedding of MSU crystals serve as a critical structural basis for tophus formation (65). Notably, the core components of tophi highly overlap with those of NETs and consist primarily of NE, MPO, antimicrobial peptides, and other bioactive substances (32, 66).

Tophi are not simply inert accumulations of urate crystals (67), but rather complex immunological defense structures with distinct structural and functional properties. Their core pathological features manifest in two main aspects: first, they display a multilayered barrier-like architecture, with MSU crystals at the core, surrounded by an outer shell composed of CD68+ macrophages, mast cells, and fibroblasts, and enriched in inflammatory mediators such as IL-1β, forming highly ordered, multicentric, and spatially heterogeneous concentric layers (68); second, they exhibit a granuloma-like structure, with surrounding aggregates of macrophages, lymphocytes, and fibroblasts that help maintain local immune homeostasis and prevent systemic spread of crystal-induced inflammation (68). Notably, although NETs exert protective negative regulatory effects during the acute phase of gout, incomplete local NET clearance or sustained excessive NET accumulation drives chronic granuloma formation (69) and promotes the development and persistence of tophi ultimately. Despite their relatively stable gross morphology, tophi represent a latent “inflammatory trigger” with a substantial risk of inflammatory reactivation. On the one hand, external stimuli—such as reduced temperature (70) and altered pH (71)—disrupt the aggNET scaffold, leading to the massive release of sequestered MSU crystals and inflammatory mediators (including IL-1β and HMGB1), which in turn trigger acute gout flares or secondary inflammatory exacerbations (70, 71). On the other hand, DNase I effectively degrades NETs and prevents their abnormal local accumulation (72, 73). However, gout patients commonly exhibit defective NET clearance, resulting in persistent retention of NETs within joints, which further promotes urate crystal deposition and tophus progression (32).

#### 3.2.2 Mechanisms of NETs in bone erosion

NETs are resistant to DNase-mediated degradation, which renders them recalcitrant to clearance and facilitates their persistent accumulation within the joint. The longer tophi persist within joint tissues, the more likely they are to induce a series of structural joint lesions, including periosteal reaction, bone erosion, cartilage damage, and osteophyte formation (34, 74). As described in Section 2.2, accumulating evidence has confirmed that the inflammatory loop formed by NETs and M1 macrophages continuously releases IL-1β and TNF-α; these inflammatory cytokines act synergistically with cytotoxic components of NETs to mediate bone erosion (75, 76). Moreover, NETs induced by MSU crystals serve as central drivers in the development and progression of gouty bone erosion, exerting their effects through multi-dimensional regulation of tissue and cellular damage. First, NETs act as a critical structural scaffold during tophus formation. Their positively charged histones anchor to the surface of MSU crystals via electrostatic interactions, while DNA fibers interweave into a three-dimensional meshwork that encases the crystals, continuously recruiting free MSU crystals from the periphery and gradually promoting the formation and deposition of large crystal clusters (32). Second, NETs directly induce damage and pathological remodeling of articular cartilage and surrounding tissues (77). Toxic components released following excessive NET accumulation exert potent cytotoxicity on bone cells (18) and upregulate the expression of matrix metalloproteinases (MMPs), further exacerbating cartilage matrix degradation and joint structural destruction (18). Third, NETs disrupt the structural stability of the cartilage extracellular matrix, rendering it more susceptible to degradation. Meanwhile, acting as DAMPs, NETs induce robust production of inflammatory cytokines via activation of the TLR4/NF-κB signaling pathway, thereby promoting chondrocyte apoptosis. These dual effects synergistically accelerate the degradation of articular cartilage tissue (78). Fourth, NETs accelerate joint erosion by inducing aberrant proliferation of fibroblast-like synoviocytes (FLSs) and directly driving osteoclastogenesis and osteoclast activation, via synovial inflammation as well as enhanced osteoclastic bone resorption (79).

**Figure 5:**
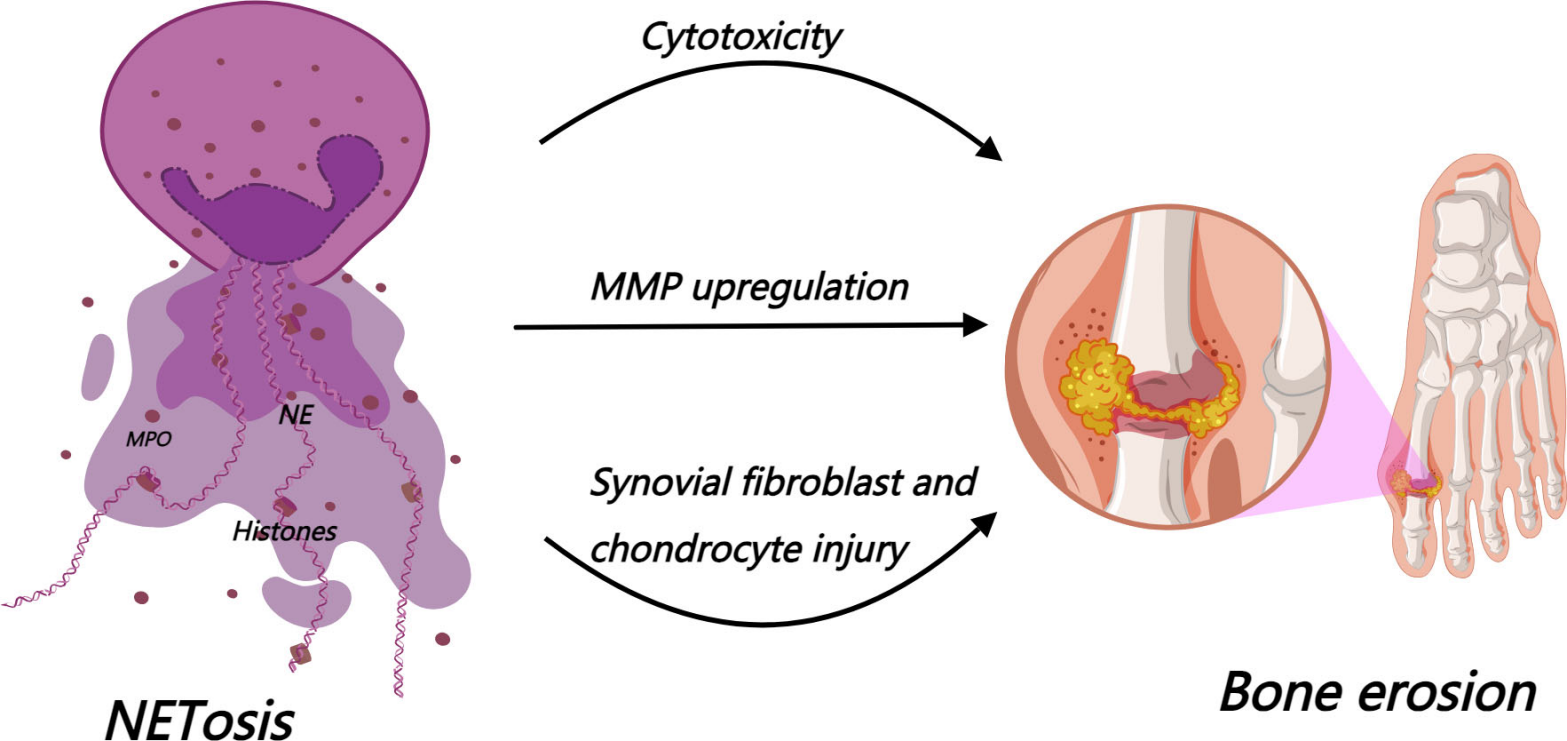
NETs promote bone erosion via cytotoxicity, MMP upregulation, and injury of synovial fibroblasts and chondrocytes.

*In vitro* experiments have further clarified the molecular mechanisms by which NETs regulate bone metabolism. NETs directly modulate the mRNA expression of alkaline phosphatase, osteoprotegerin (OPG), and receptor activator of nuclear factor-κB ligand (RANKL) in osteoblasts. Additionally, via osteoblast-mediated paracrine signaling, NETs indirectly upregulate osteoclast differentiation markers. Ultimately, through dysregulation of the OPG/RANKL/RANK axis, NETs promote osteoclast differentiation and activation, thereby participating in bone destruction via both direct and indirect pathways (18) (**Figure 5**). Animal models have validated the osteoprotective effects of targeting NETs. Degradation of NETs by DNase I (43) or inhibition of NE (62) both significantly attenuate bone destruction, highlighting the potential therapeutic value of targeting NETs in alleviating gouty bone erosion.

## 4 Potential value of nets as diagnostic and therapeutic markers in gout

Researchers have proposed NET-related molecules as potential biomarkers based on the above mechanisms. Serum levels of cell-free DNA, MPO-DNA complexes, and citrullinated histone H3 (CitH3) are significantly elevated in patients with gout and are associated with disease activity and flare frequency (80). Clinical studies have confirmed that NET levels in the synovial fluid of patients with gout are positively correlated with MSU crystal burden. Additionally, serum cell−free DNA concentrations are closely associated with flare frequency and disease chronicity, suggesting their potential as biomarkers of disease activity (65, 80). Of note, although circulating NET levels are higher in gout patients than in healthy controls, they do not associate with disease activity or inflammation (80). Particularly during the initial phase of urate-lowering therapy (ULT), the partial dissolution of the tophus destabilizes the aggNET structure, exposing previously sequestered crystals to nearby innate immune cells, and the subsequent release of MSU crystals from tophus dissolution can readily trigger renewed NET formation, leading to gout “flares” (62). In this context, dynamic changes in serum cell-free DNA may serve as a biomarker for assessing therapeutic response and predicting the risk of inflammatory flare recurrence.

However, insufficient specificity currently limits the clinical application and research of NET-related biomarkers; such markers are elevated in various inflammatory conditions, including infection, trauma, and autoimmune diseases, and thus cannot specifically reflect NET formation (8). Meanwhile, conventional NET detection methods suffer from technical drawbacks. Sytox Green can rapidly penetrate viable cells and generate nonspecific fluorescent signals. At the same time, MPO antibody staining may produce residual fluorescence due to nonspecific binding of granular proteins to cell membranes. Both of these issues lead to inaccurate quantification of NETosis. Although the development of PLaNET—a novel fluorescent polymer—has effectively overcome the nonspecific signal limitations of traditional assays by specifically binding to extracellular chromatin released from neutrophils, enabling the specific and standardized quantification of NETosis (81), this reagent is limited in its application scope. It cannot adapt for kinetic detection of NETosis in microplate-based systems. De et al. (82) reported that histone H3 N-terminal cleavage serves as a specific marker for identifying the cleavage of the H3 N-terminus during NET formation, in contrast to conventional markers such as citrullinated H3 (CitH3) or extracellular DNA alone. This marker is more effective than traditional methods, as it detects a larger proportion of NETs and can identify NET formation at early stages of NETosis. However, H3 N-terminal cleavage is typically coupled to ROS and PAD4 activation, and NETosis induced by specific stimuli may not elicit it. Collectively, the classification and quantification of NETs still lack techniques with sufficient specificity, stability, and versatility across experimental settings. Further optimization of detection systems and the development of novel technologies are urgently needed.

## 5 Potential value of NETs-targeted therapy in gout

Given that NETs are widely involved in the pathogenesis of various inflammatory diseases, including gout, the development of NET−targeted therapeutic strategies to alleviate their pathological damage has been recognized as a major research focus and an urgent requirement in this field. For the inhibition of NET formation, PAD4 inhibitors represent a central therapeutic direction. Cl-amidine reduces NET release by blocking the core step of histone citrullination described in Section 2.1, and has proven effective in alleviating joint inflammation in animal models (43). BB-Cl-pyrimidine exhibits improved bioavailability and suppresses PAD4 activity while preserving NOX2-mediated innate immune functions (83). GSK484—a highly specific, reversible PAD4 inhibitor—selectively targets key molecules within this signaling pathway without impairing host antiviral immune defense (84, 85), thereby further supporting the feasibility of translating PAD4 inhibitors into clinical practice. However, the role of NETs in antibacterial defense is distinctly different. PAD4-deficient mice fail to form functional NETs, resulting in reduced killing of *Staphylococcus aureus* and increased susceptibility to necrotizing fasciitis (86). It should be noted that the biological effects of NETs are highly dependent on disease stage and pathological context. In bacterial infection-related conditions such as sepsis, NETs serve as a critical defensive barrier against pathogens during the early phase of the disease, and inhibiting NETs at this stage significantly increases bacterial burden (87), potentially exacerbating disease outcomes. Therefore, long-term use of PAD4 inhibitors (e.g., GSK484) may elevate the risk of bacterial infections, necessitating routine monitoring of complete blood counts and infection indicators (88). Precise therapeutic strategies should be tailored to the different stages of disease progression. Moreover, caution is warranted in clinical applications, as such drugs have not yet entered human clinical trials, and their translational potential and clinical implementation require further investigation.

Humanized monoclonal antibodies offer a more specific approach for NET-targeted intervention, with CIT-013—the first monoclonal antibody targeting citrullinated histones—serving as a representative example (89). As of September 2025, researchers have administered the first patient in a Phase II clinical trial of this drug, marking the formal entry of NET-targeted antibody therapies into late-stage human clinical trials. In addition, several widely used clinical therapeutics have been identified and repurposed as NET inhibitors. For example, colchicine—beyond its canonical chemotaxis-inhibitory effects—has been demonstrated to suppress NET formation in preclinical animal studies, with efficacy comparable to that of the PAD4 inhibitor Cl-amidine (90). Although clinicians commonly use allopurinol and febuxostat as urate-lowering therapies, preclinical studies have shown that these agents also modulate NET formation by reducing MPO-DNA complex formation and decreasing levels of citrullinated histone H3 (CitH3) (91, 92), thereby revealing their potential immunomodulatory properties. Cyclosporine A effectively suppresses NET formation by reducing ROS production and IL-8 secretion (93).

To enhance NET clearance, exogenous DNase I is the most extensively studied agent, which directly degrades the DNA backbone of NETs and structurally disrupts these extracellular traps (43, 72). Recent studies have indicated the therapeutic potential of DNase I in rat models of intestinal ischemia-reperfusion injury (94) and initiated clinical trials for acute respiratory distress syndrome secondary to COVID-19 (95). Related studies have also demonstrated a significant imbalance between NET formation and DNase activity or abundance in severe and critical COVID-19 patients (96), providing a strong theoretical basis for the application of DNase I in NET-related disorders. Dual-activity DNase I mutants developed on this basis show significantly higher degradation efficiency toward double-stranded DNA (dsDNA) and chromatin than native DNase I, and exhibit favorable stability in the mouse circulatory system (97), representing a promising direction for optimizing NET clearance strategies.

Natural compounds provide diverse candidates for NET-targeted therapy. Astilbin inhibits NET formation by blocking the P2Y6 receptor–IL-8/CXCR2 signaling pathway and downregulating CitH3 expression (98). Plant-derived compounds such as sinomenine and palmatine can directly block NETosis by inhibiting the core molecular activity of the NLRP3/IL-1β pathway described in Section 2.2, thereby alleviating local inflammatory responses in gout (99, 100). Collectively, substantial advances in NET-targeted therapies have led to a dual intervention strategy that both inhibits NET formation and promotes NET clearance. This strategy encompasses novel targeted agents, repurposed clinical therapeutics, and natural bioactive compounds (**Table 1**). However, further basic and clinical studies are still required to validate the efficacy and safety of such agents in specific diseases, including gout, to optimize precise dosing regimens across different disease stages, and to improve the *in vivo* targeting and stability of novel formulations. Researchers still need to fully investigate the clinical translation and translational application of these therapeutic strategies.

**Table 1:** Summary of targeted intervention strategies for NET.

Intervention direction	Drug/preparation name	Mechanism	Research evidence	Reference
Inhibition of NETs formation (PAD4 inhibitors)	Cl-amidine	Blocks histone citrullination	Animal model	(43)
	BB-Cl-pyrimidine	Inhibits PAD4 activity and maintains NOX2-mediated innate immune function	Animal model	(83)
	GSK484	Highly specific and reversible inhibition of PAD4	Cell experiments in humans and animals	(84)
Inhibition of NETs formation (humanized monoclonal antibody)	CIT-013 monoclonal antibody	Targets citrullinated histones	First patient dosed in Phase II clinical trial	(89)
Inhibition of NETs formation (repurposed drugs)	Colchicine	Inhibits NETs formation in addition to its classic chemotaxis-inhibiting effect	Animal model	(90)
	Allopurinol	Inhibits superoxide production	Cell experiments in animals	(91)
	Febuxostat	Reduces CitH3 expression	Animal model	(92)
	Cyclosporin A	Reduces ROS production and IL-8 secretion	Clinical trial	(93)
Inhibition of NETs formation (natural compounds)	Astilbin	Blocks the P2Y6 receptor-IL-8/CXCR2 signaling pathway and downregulates CitH3 expression	Cell experiments and animal models	(98)
	Sinomenine	Inhibits the activity of core molecules in the NLRP3/IL-1β pathway	Cell experiments and animal models	(99)
	Palmatine	Inhibits the activity of core molecules in the NLRP3/IL-1β pathway	Cell experiments and animal models	(100)
Promotion of NETs clearance	Exogenous DNase I	Directly degrades the DNA backbone of NETs	Animal models and Clinical trials	(94–96),
	Dual-activity DNase I mutant	Exhibits higher efficiency in degrading dsDNA and chromatin than native DNase I	Animal model	(97)

## 6 Conclusion and perspective

In summary, this review highlights several key findings regarding the dual role of NETs in gout. During the acute phase, NETs help limit inflammation by forming aggNETs that sequester MSU crystals and degrade inflammatory mediators; however, they can also amplify inflammatory cascades via complement and inflammasome activation. In the chronic phase, impaired clearance leads to persistent NET accumulation, which serves as a structural scaffold for tophus formation and promotes bone erosion by disrupting the bone metabolic axis. Thus, the NET function varies with disease stage and local microenvironment. Regarding the mechanisms of NET formation, three main types of NETosis have been identified—suicidal, vital, and mitochondrial DNA release—with MSU crystals primarily triggering ROS-dependent suicidal NETosis, while alternative pathways (e.g., Src/Syk and RIPK1-RIPK3-MLKL signaling) are also involved. Notably, all major types of NETosis begin with PAD4-mediated histone citrullination, a critical initiating step. Finally, In gout, inflammatory regulation and tissue damage involve complex interactions among multiple pathways and cell types. Positive feedback loops develop between NETs, macrophages, and synovial fibroblasts, while internal resolution mechanisms—including immune silencing by aggNETs—are also activated. NETs contribute to bone erosion through several methods: directly damaging bone cells, disrupting the OPG/RANKL/RANK axis, and increasing cartilage matrix breakdown. Precisely modulating the balance between NET formation and clearance to achieve two key therapeutic goals—controlling acute inflammation while promoting tophus dissolution and bone repair during the chronic phase—remains a major challenge in gout management. Further studies are warranted to dissect the dynamic regulatory mechanisms of NETs across distinct stages of gout and to develop targeted intervention strategies with high specificity and stage adaptability. Such advances will drive the evolution of gout management from symptomatic control toward the restoration of immune homeostasis. To achieve this transition, researchers must overcome three major bottlenecks: First, they must comprehensively delineate the dynamic evolutionary patterns of NETs at different disease stages. Second, they must establish organoid or microfluidic models that recapitulate human pathological features, thereby enabling visual tracking of the entire NET formation-clearance cycle. Third, they must implement precision diagnostic and therapeutic systems based on NET subtyping in clinical translation, integrating dynamic imaging, liquid biopsies, and functional assays to guide personalized interventions. Only through these approaches can the field shift from global immunosuppression to stage-specific immunomodulation, transforming the double-edged sword of NETs into a controllable therapeutic tool and opening new avenues for the management of gout and other autoimmune disorders.
